# Prognostic Factors of Survival in Pancreatic Cancer Metastasis to Liver at Different Ages of Diagnosis: A SEER Population-Based Cohort Study

**DOI:** 10.3389/fdata.2021.654972

**Published:** 2021-09-27

**Authors:** Meiqi Liu, Moran Wang, Sheng Li

**Affiliations:** ^1^ Department of Infectious Disease, Tongji Hospital, Tongji Medical College, Huazhong University of Science and Technology, Wuhan, China; ^2^ Department and Institute of Infectious Disease, Xi’an Children’s Hospital, Xi’an Jiaotong University, Xi’an, China; ^3^ Department of Cardiology, Tongji Hospital, Tongji Medical College, Huazhong University of Science and Technology, Wuhan, China

**Keywords:** pancreatic cancer, liver, prognosis, metastasis, treatment

## Abstract

**Background:** Liver is a common metastatic organ for most malignancies, especially the pancreas. However, evidence for prognostic factors of pancreatic cancer metastasis to the liver at different ages is lacking. Thus, we aimed to evaluate the predictors of patients with pancreatic cancer metastasis to liver grouped by age of diagnosis.

**Methods:** We chose the patients diagnosed between 2004 and 2015 from the SEER database. The primary lesions of metastatic liver cancer between sexes were compared using the Pearson’s chi-square test for categorical variables. The overall survival (OS) and cancer-specific survival (CSS) were the endpoint of the study. The prognostic factors were analyzed with the Kaplan-Meier method and log-rank test, and Cox proportional-hazards regression model.

**Results:** The main primary sites of metastatic liver cancer for our patients are lung and brunchu, sigmoid colon, pancreas, which in males are lung and bronchu, sigmoid colon and pancreas, while breast, lung and bronchu, sigmoid colon in females. Furthermore, we explored the prognostic factors of pancreatic cancer metastasis to liver grouped by age at diagnosis. Tumor grade, histology and treatment are valid prognostic factors in all age groups. Additionally, gender and AJCC N stage in age<52 years old, while race and AJCC N stage in age >69 years old were predictors. Surgery alone was the optimal treatment in group age>69 years old, whereas surgery combined with chemotherapy was the best option in the other groups.

**Conclusion:** Our study evaluated the predictors of patients with pancreatic cancer metastasis to liver at various ages of diagnosis.

## Introduction

The liver is the most frequently afflicted metastatic organ second to the lymph nodes for most malignancies (Jaques et al., 1995; Hess et al., 2006; Amankwah et al., 2013; Ryu et al., 2013). The most common tumors with liver metastases arise from the portal venous drainage system, which provides about two-thirds of the liver’s blood supply. Because lesions are usually asymptomatic, liver involvement in metastasis is often neglected and poorly studied, and even extensive infiltration of metastatic tumor may not alter its function or homeostasis until late in the disease (Clark et al., 2016). There are few epidemiological studies on metastatic liver cancer, but 30–70% of patients die of liver metastasis (Pickren J and Lane, 1982) and most patients with liver metastases will die of the primary disease (Gilbert Ha et al., 1982).

As one of the deadliest malignant tumors in the world (Ferlay et al., 2015; Schild and Vokes, 2016), pancreatic cancer is the eighth most common cause of cancer in males and the sixth most common cause of cancer in females. In decades, a large number of studies have shown that the development of pancreatic cancer was closely related to age. The aging trend of the population in the world is challenging the current treatments and caring for patients with pancreatic cancer (Bray et al., 2018; Ferlay et al., 2018). The underlying mechanisms of pancreatic cancer is complicated and uncertain, accompanied with poor prognosis (Maisonneuve, 2019). According to the original site in pancreas, pancreatic cancer is classified as endocrine and exocrine pancreatic cancer, and the latter is more common and has a higher risk of mortality in both females and males (Fesinmeyer et al., 2005). Additionally, the majority of exocrine pancreatic cancer is adenocarcinoma (Li, 2001; Cowgill and Muscarella, 2003). Approximately 50% of pancreatic cancer patients are diagnosed with distant metastases (Mayo et al., 2012), and the most common site of distal metastases found at autopsy was the liver, followed by the peritoneum, lungs and pleura, bones, and adrenal glands (Kamisawa et al., 1995; Mao et al., 1995; Embuscado et al., 2005; Disibio and French, 2008). Previous studies suggested risk factors of pancreatic cancer involving smoking, positive family history and genetics, diabetes, obesity, dietary factors, alcohol consumption, and physical inactivity (Yadav and Lowenfels, 2013; Ilic and Ilic, 2016). Age, race, tumor size, grade, lymph node metastasis (Mayo et al., 2012), AJCC stage (Kamarajah et al., 2017) and treatment (Ansari et al., 2019) are also reported associated with the survival of pancreatic cancer patients. However, evidence for prognostic factors in pancreatic cancer with distant metastasis is rare. However, evidence for prognostic factors in pancreatic cancer with distant metastasis is rare. Moreover, Andrew A et al. and previous studies reported that treatment strategies for pancreatic cancer differentiate in diverse range of ages (Wheeler and Nicholl, 2014). Thus, the objective of this study is to determine the differences in primary sites of metastatic liver cancer between males and females. Furthermore, we evaluated the prognostic risk factors of pancreatic cancer metastasis to liver at different ages of diagnosis through the Cox regression model.

## Materials and Methods

### Data Source

The data was from the National Cancer Institute’s Surveillance, Epidemiology, and End Results (SEER) program between 2004 and 2015. The program contains the population-based central cancer registries of 18 geographically defined regions. Because all the data used in the study was retrieved from the SEER database with publicly available methods, the study did not require local moral approval or a declaration.

### Patient Selection

The inclusion criteria included: 1) The disease was diagnosed between 2004 and 2015; 2) metastases of the primary tumor were at the liver; 3) there was only one primary tumor; 4) the diagnosis of the disease was histologically positive; 5) there were more than 0 days of survival.

**FIGURE 1:**
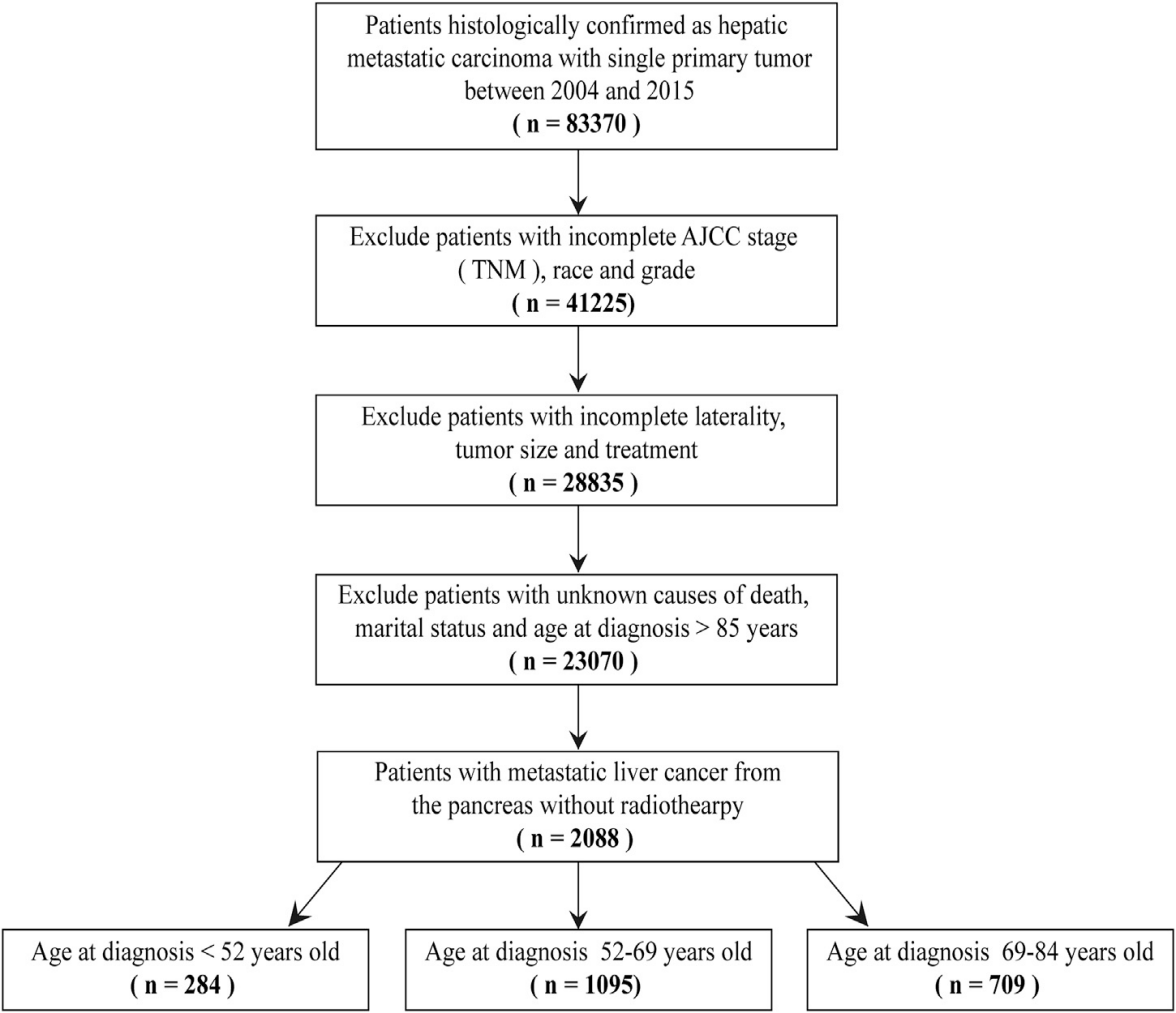
Study cohort.

The exclusion criteria included: 1) age≥85 years old; 2) the demographics of patients were incomplete, including race and marital status; 3) the clinicopathological characteristics of patients were incomplete, including grade, AJCC seventh stage (TNM), tumor size, laterality, causes of death and treatment methods; 4) patients treated with radiotherapy; 5) the type of reporting source was autopsy only or death certificate only. (Figure 1).

We used the histopathology codes from the International Classification of Disease for Oncology third edition (ICD-O-3) to define the primary sites of patients with hepatic metastatic carcinoma. In the ICD-O-3, the codes were defined as follows: code 19-29 (tongue), code 50-69 (gum and other mouth), code 70-89 (salivary gland), code 90-99 (tonsil), code 110-119 (nasopharynx), code 129-139 (hypopharynx), code 150-159 (Esophagus), code 160-169 (stomach), code 170-179 (small intestine), code 180 (cecum), code 181 (appendix), code 182 (ascending colon), code 183 (hepatic flexure), code 184 (transverse colon), code 185 (splenic flexure), code 186 (descending colon), code 187 (sigmoid colon), code 199 (rectosigmoid junction), code 209 (rectum), code 210-218 (Anus, Anal Canal and Anorectum), code 220 (liver), code 221 (intrahepatic bile duct), code 239 (gallbladder), code 240-241 (other biliary), code 250-259 (pancreas), code 300-319 (nose, nasal cavity and middle ear), code 320-329 (larynx), code 340-349 (lung and bronchu), code 380, 472-479, 490-499 (soft tissue including heart), code 381-383 (trachea, mediastinum and other respiratory organs), code 384 (pleura), code 400-419 (bones and joints), code 440-449 (skin excluding basal and squamous), code 480 (retroperitoneum), code 481-482 (peritoneum, omentum and mesentery), code 500-509 (breast), code 510-519 (vulva), code 529 (vagina), code 530-539 (cervix uteri), code 540-549 (corpus uteri), code 569 (ovary), code 570 (other female genital organs), code 601 (penis), code 619 (prostate), code 620-629 (testis), code 649-659 (kidney and renal pelvis), code 669 (ureter), 670-679 (urinary bladder ), code 739 (thyroid) and code 740-755 (other endocrine including thymus).

According to the age at diagnosis of patients, we divided them into three groups, including age at diagnosis <52 years old, age at diagnosis 52–69 years old and age at diagnosis 69–84 years old.

### Clinical Variables of Patients

Information on demographic factors (age, race, sex and marital status), tumor-related factors (tumor size, grade, histology and AJCC TNM staging system), therapeutic factors (surgery and chemotherapy) and follow-up were collected from the SEER database. And follow-up period ended in 2015. Based on the Surgery Codes of the SEER program and information about other treatments, we divided the treatment options into categories: no treatment (N), surgery alone (S), chemotherapy alone (C), surgery combined with chemotherapy (SC).

OS and CSS were the interesting endpoint, and the cancer-specific death was based on the code of “SEER cause-specific death classification” in the SEER database. OS was measured from the date on which the first-time definite diagnosis was made until the date of death caused by any cause or the most recent follow-up.

### Statistical Analysis

Age and tumor size are categorized according to the best cut-off value produced by the x-tile software version 3.6.1 (Yale University School of Medicine, US). (S2) The incidence rates were calculated by using R software. And baseline patients’ demographics and clinicopathological characteristics were compared using the Pearson’s chi-square test for categorical variables. The independent risk factors were identified by univariate and multivariate Cox proportional-hazards regression analyses for OS. R software version 4.0.2 (R Project, Vienna, Austria) was used for all analysis. Statistically significant cutoff value was set up as *p* < 0.05, two-sided. *p* < 0.2 was selected as filter value for univariate to multivariate analysis.

## Results

### The Frequency Distribution of Primary Lesions of Metastatic Liver Cancer

**FIGURE 2:**
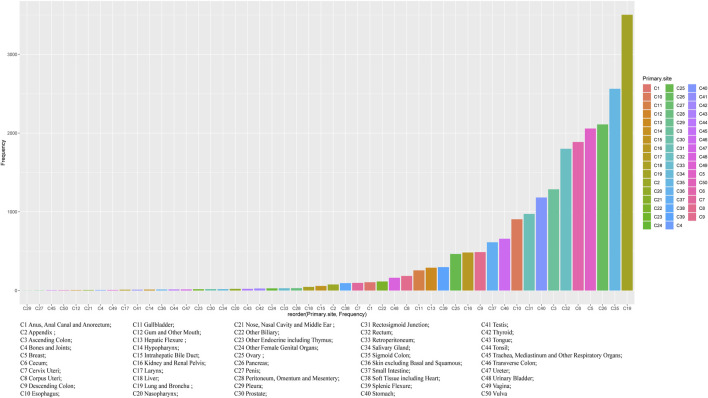
Frequency Distribution of primary tumour sources of hepatic metastatic carcinoma.

**FIGURE 3:**
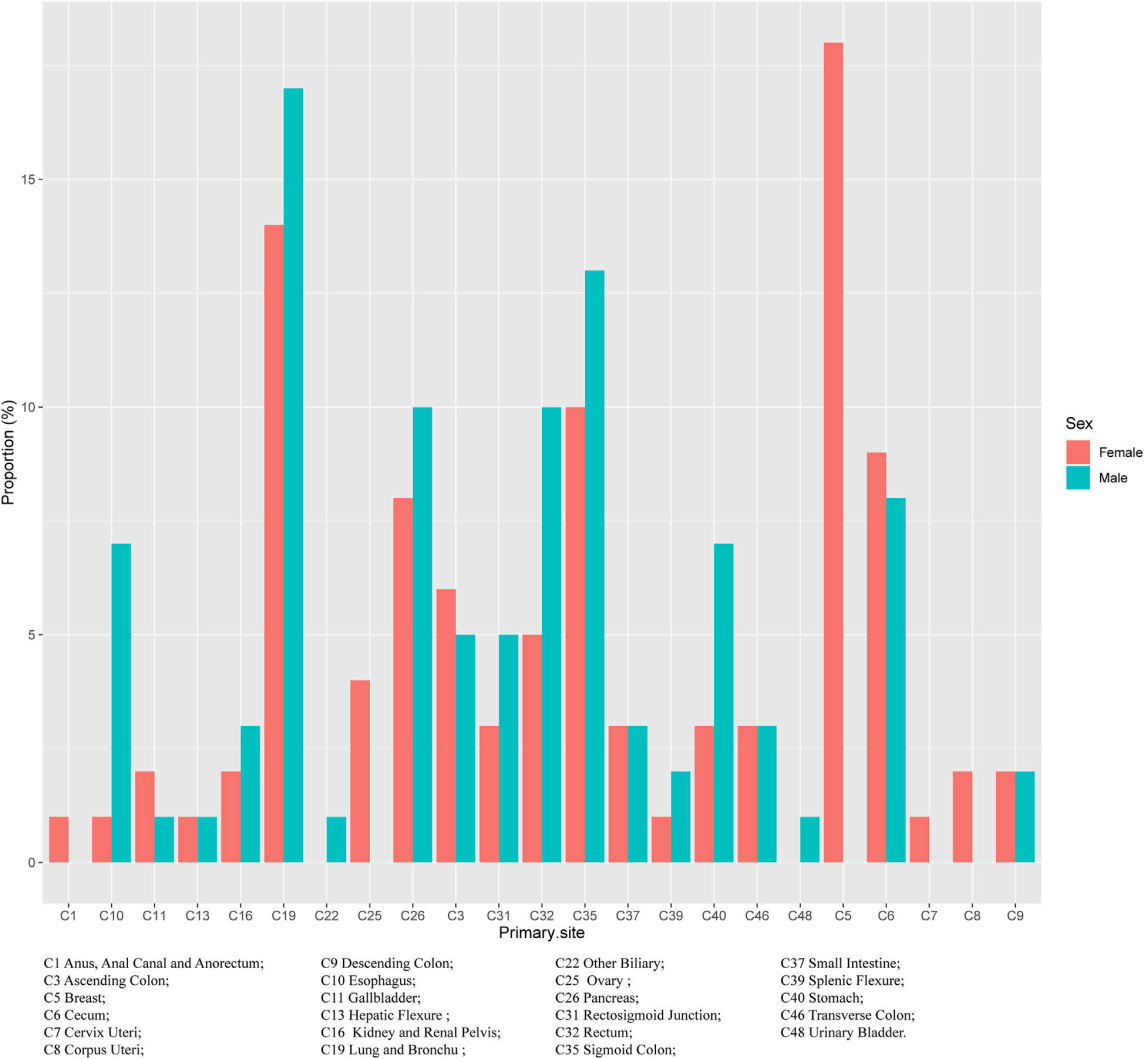
The purpose of primary tumour sources of liver metastatic carcinoma in both sexes.

Regardless of gender, the most common primary site of hepatic metastatic carcinoma was lung and brunchu that accounted for 15.18% of all primary lesions, followed by sigmoid colon (11.11%), pancreas (9.15%), breast (8.92%), cecum (8.18%) and rectum (7.81%). The result of Pearson’s chi-square test showed that the primary sites of hepatic metastatic carcinoma were significantly different between males and females including anus, anal canal and anorectum (*p* < 0.001), ascending colon (*p* < 0.05), breast (*p* < 0.001), cervix uteri (*p* < 0.001), corpus colon (*p* < 0.001), descending colon (*p* < 0.05), esophagus (*p* < 0.001), gallbladder (*p* < 0.001), hepatic flexure (*p* < 0.05), kidney and renal pelvis (*p* < 0.001), larynx (*p* < 0.05), liver (*p* < 0.001), lung and bronchus (*p* < 0.001), nasopharynx (*p* < 0.01), other female genital organs (*p* < 0.001), ovary (*p* < 0.001), pancreas (*p* < 0.001), peritoneum, omentum and mesentery (*p* < 0.001), prostate (*p* < 0.001), rectosigmoid junction (*p* < 0.001), rectum (*p* < 0.001), sigmoid colon (*p* < 0.001), splenic flexure (*p* < 0.001), stomach (*p* < 0.001), testis (*p* < 0.01), urinary bladder (*p* < 0.001) and vulva (*p* < 0.05). (Table 1). In females, the top five most common primary lesions of hepatic metastases were breast (18.39%), lung and bronchu (13.51%), sigmoid colon (9.55%), cecum (8.51%) and pancreas (8.17%), while in males were lung and bronchu (16.75%), sigmoid colon (12.56%), pancreas (10.06%), rectum (10.02%) and cecum (7.87%). (Figures 2,3).

**TABLE 1:** The frequency distribution of primary lesions of metastatic liver cancer.

Primary.site, n (%)	Total (*n* = 23,070)	Female (*n* = 11,139)	Male (*n* = 11,931)	*p* Value
Anus, Anal Canal and Anorectum	105 (0.46)	72 (0.65)	33 (0.28)	<0.001***
Appendix	77 (0.33)	42 (0.38)	35 (0.29)	>0.05
Ascending Colon	1,286 (5.57)	656 (5.89)	630 (5.28)	<0.05*
Bones and Joints	6 (0.03)	1 (0.01)	5 (0.04)	>0.05
Breast	2058 (8.92)	2048 (18.39)	10 (0.08)	<0.001***
Cecum	1887 (8.18)	948 (8.51)	939 (7.87)	>0.05
Cervix Uteri	97 (0.42)	97 (0.87)	0 (0)	<0.001***
Corpus Uteri	185 (0.80)	185 (1.66)	0 (0)	<0.001***
Descending Colon	488 (2.12)	210 (1.89)	278 (2.33)	<0.05*
Esophagus	905 (3.92)	115 (1.03)	790 (6.62)	<0.001***
Gallbladder	256 (1.11)	183 (1.64)	73 (0.61)	<0.001***
Gum and Other Mouth	5 (0.02)	2 (0.02)	3 (0.03)	>0.05
Hepatic Flexure	290 (1.26)	120 (1.08)	170 (1.42)	<0.05*
Hypopharynx	12 (0.05)	2 (0.02)	10 (0.08)	>0.05
Intrahepatic Bile Duct	58 (0.25)	28 (0.25)	30 (0.25)	>0.05
Kidney and Renal Pelvis	483 (2.09)	180 (1.62)	303 (2.54)	<0.001***
Larynx	10 (0.04)	1 (0.01)	9 (0.08)	<0.05*
Liver	46 (0.20)	6 (0.05)	40 (0.34)	<0.001***
Lung and Bronchu	3,503 (15.18)	1,505 (13.51)	1998 (16.75)	<0.001***
Nasopharynx	21 (0.09)	3 (0.03)	18 (0.15)	<0.01**
Nose, Nasal Cavity and Middle Ear	6 (0.03)	4 (0.04)	2 (0.02)	>0.05
Other Biliary	115 (0.50)	49 (0.44)	66 (0.55)	>0.05
Other Endocrine including Thymus	16 (0.07)	49 (0.05)	10 (0.08)	>0.05
Other Female Genital Organs	27 (0.12)	27 (0.24)	0 (0)	<0.001***
Ovary	464 (2.01)	464 (4.17)	0 (0)	<0.001***
Pancreas	2,110 (9.15)	910 (8.17)	1,200 (10.06)	<0.001***
Penis	2 (0.01)	0 (0)	2 (0.02)	>0.05
Peritoneum, Omentum and Mesentery	30 (0.13)	27 (0.1)	3 (0.15)	<0.001***
Pleura	1 (0.00)	0 (0)	1 (0.01)	>0.05
Prostate	18 (0.08)	0 (0)	18 (0.15)	<0.001***
Rectosigmoid Junction	973 (4.22)	384 (3.45)	589 (4.94)	<0.001***
Rectum	1801 (7.81)	605 (5.43)	1,196 (10.02)	<0.001***
Retroperitoneum	29 (0.13)	11 (0.10)	18 (0.15)	>0.05
Salivary Gland	18 (0.08)	7 (0.06)	11 (0.09)	>0.05
Sigmoid Colon	2,563 (11.11)	1,064 (9.55)	1,499 (12.56)	<0.001***
Skin excluding Basal and Squamous	13 (0.06)	5 (0.04)	8 (0.07)	>0.05
Small Intestine	613 (2.66)	284 (2.55)	329 (2.76)	>0.05
Soft Tissue including Heart	94 (0.41)	54 (0.48)	40 (0.34)	>0.05
Splenic Flexure	297 (1.29)	113 (1.01)	184 (1.54)	<0.001***
Stomach	1,182 (5.12)	329 (2.95)	853 (7.15)	<0.001***
Testis	10 (0.04)	0 (0)	10 (0.08)	<0.01**
Thyroid	26 (0.11)	14 (0.13)	12 (0.10)	>0.05
Tongue	22 (0.10)	6 (0.05)	16 (0.13)	>0.05
Tonsil	14 (0)	4 (0)	10 (0)	>0.05
Trachea, Mediastinum and Other Respiratory Organs	4 (0.02)	0 (0)	4 (0.03)	>0.05
Transverse Colon	658 (2.85)	302 (2.71)	356 (2.98)	>0.05
Ureter	14 (0.06)	7 (0.06)	7 (0.06)	>0.05
Urinary Bladder	162 (0.70)	49 (0.44)	113 (0.95)	<0.001***
Vagina	6 (0.03)	6 (0.05)	0 (0)	<0.05*
Vulva	4 (0.02)	4 (0.04)	0 (0)	>0.05

*, two-sided *p* values <0.05; **, two-sided *p* values <0.01; ***, two-sided *p* values <0.001.

### The Effect of Age at Diagnosis With Pancreatic Cancer Metastasis to Liver

**FIGURE 4:**
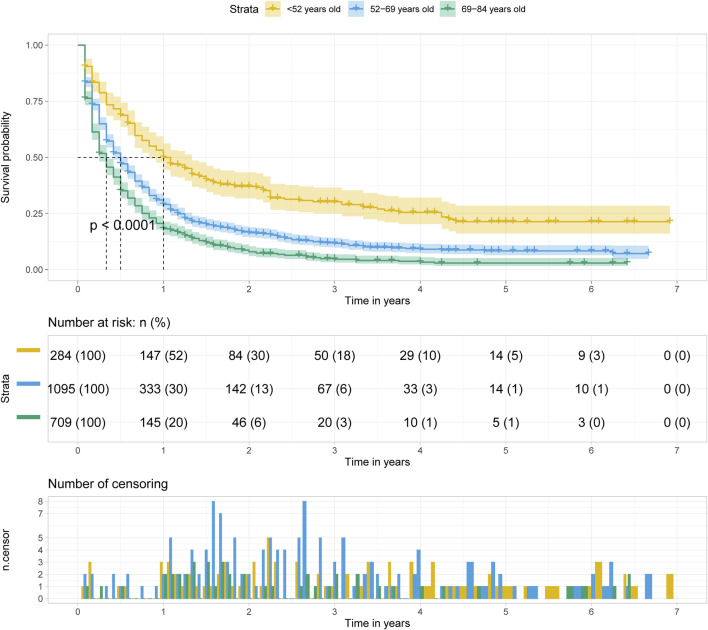
Kalpan Meier survival curve showing the effect of age at diagnosis with pancreatic cancer metastasis to liver.

The Kaplan Meier survival curve showed significant difference in overall survival for patients diagnosed at different age groups (*p* < 0.001). The overall survival time was negatively correlated with the age at diagnosis. Among the three groups, the prognosis of patients diagnosed at age less than 52 years old was the best, and of which the median survival time was 1 year. (Figure 4).

### The Effect of Treatment with Pancreatic Cancer Metastasis to Liver

**FIGURE 5:**
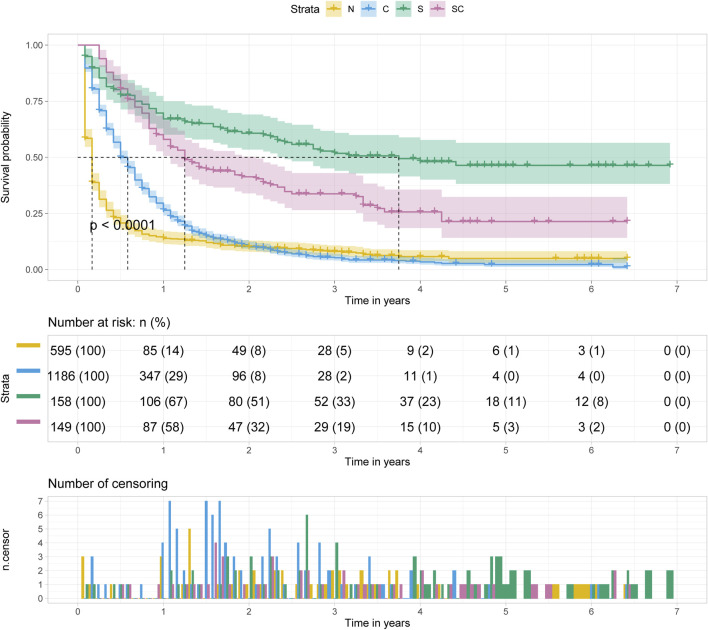
Kalpan Meier survival curve showing the effect of treatment with pancreatic cancer metastasis to liver.

Regardless of age at diagnosis, surgery alone (S) was the optimal treatment option for patients with pancreatic cancer metastasis to liver, followed by surgery combined with chemotherapy (SC), chemotherapy alone (C) and no treatment (N) (*p* < 0.001). And the median survival time of patients with surgery alone was approximately 3.5–4 years. (Figure 5).

### The Relative Hazard Ratio of Treatment and Age at Diagnosis

**FIGURE 6:**
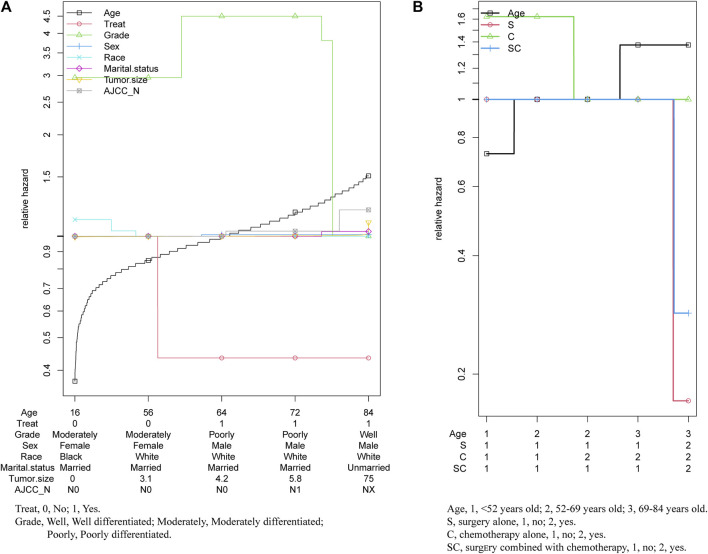
Relative hazard ratio of multivariables in patients with pancreatic cancer metastasis to liver.

As the multivariable hazard ratio of in prognosis displayed in Figure 6, with the increase of the age of diagnosis, treatment showed significantly protective effect, while grade had a significant effect on prognosis only in younger age. And other prognostic factors had almost no significant change. (Figure 6A). Thus, we further analyzed the relative hazard ratio of diverse treatment options and age of diagnosis in patients, we found that when patients were diagnosed at a younger age, chemotherapy alone was the most adverse risk factor, while when diagnosed at an older age, age at diagnosis was the most adverse risk factor for the outcome. What’s more, for patients diagnosed at all ages, chemotherapy alone was the treatment with the worst effect on prognosis, while for patients diagnosed at age more than 69 years old, surgery was better than combined with chemotherapy. (Figure 6B).

### Clinical Characteristics of the Patients With Pancreatic Cancer Metastasis to Liver

Demographic characteristics of 2088 patients with pancreatic cancer metastasis to liver grouped by age at diagnosed during the 12-years study period (between 2004 and 2015) in the SEER database are shown in Table 2. In this study, sex (*p* = 0.002), race (*p* = 0.031), marital status (*p* < 0.001), tumor grade (*p* < 0.001), AJCC N stage (*p* = 0.005), treatment (*p* < 0.001), median survival time (*p* < 0.001) and vital status (*p* < 0.001) were the parameters with significant difference among different groups. On the whole, most patients were married white males whose tumors were poorly differentiated and less than 4.9 cm in size, treated with chemotherapy alone (C). The most common histological type of tumors was adenomas and adenocarcinomas. Compared with the other groups, well differentiated tumors (25%), surgery alone (S, 14.44%) or surgery combined with chemotherapy (SC, 11.27%) for treatment strategies and longer survival time (12 months) would more likely to occur in age <52 years old group.

**TABLE 2:** Clinical characteristics of the patients with pancreatic cancer metastasis to liver grouped by age at diagnosis.

Variables	Total (*n* = 2088)	<52 years old (*n* = 284)	52–69 years old (*n* = 1095)	69-84 years old (*n* = 709)	*p* value
Sex, n (%)	—	—	—	—	0.002**
Female	900 (43.10)	119 (41.90)	438 (40.00)	343 (48.38)	—
Male	1188 (56.90)	165 (58.10)	657 (60.00)	366 (51.62)	—
Race, n (%)	—	—	—	—	0.031*
White	1,651 (79.07)	207 (73.89)	865 (79.00)	579 (81.66)	—
Black	261 (12.50)	44 (15.49)	143 (13.06)	74 (13.06)	—
Other	176 (8.43)	33 (11.62)	87 (7.95)	56 (7.90)	—
Marital status, n (%)	—	—	—	—	<0.001***
Unmarried	333 (15.95)	87 (30.63)	186 (16.99)	60 (8.46)	
Married	1755 (84.05)	197 (69.37)	909 (83.01)	649 (91.54)	
Grade, n (%)	—	—	—	—	<0.001***
Well differentiated	257 (12.31)	73 (25.70)	108 (9.86)	76 (10.72)	—
Moderately differentiated	756 (36.21)	82 (28.87)	414 (37.81)	260 (36.67)	—
Poorly differentiated	1,000 (47.89)	116 (40.85)	539 (49.22)	345 (48.66)	—
Undifferentiated	75 (3.59)	15 (4.58)	34 (3.95)	28 (3.95)	—
Tumor size, n (%)	—	—	—	—	0.325
<4.9 cm	1,285 (61.54)	164 (57.75)	669 (61.10)	452 (63.75)	
4.9–7.4 cm	565 (27.06)	83 (29.33)	294 (26.85)	188 (26.52)	
>7.4 cm	238 (11.40)	37 (13.03)	132 (12.05)	69 (9.73)	
AJCC N, n (%)	—	—	—	—	<0.005**
N0	1,074 (51.44)	129 (45.42)	544 (49.68)	401 (56.56)	
N1	804 (38.51)	129 (45.42)	432 (39.45)	243 (34.27)	
NX	210 (10.06)	26 (9.15)	119 (10.87)	65 (9.17)	
Histology, n (%)	—	—	—	—	0.935
Adenomas and adenocarcinomas	1,688 (80.84)	225 (79.23)	896 (81.83)	567 (79.97)	—
Ductal and lobular neoplasms	166 (7.95)	25 (8.80)	82 (7.49)	59 (8.32)	—
Epithelial neoplasms	116 (5.56)	16 (5.63)	60 (5.48)	40 (5.64)	—
Others	118 (5.65)	18 (6.34)	57 (5.21)	43 (6.06)	—
Treat n (%)	—	—	—	—	<0.001***
N	595 (28.50)	51 (17.96)	273 (24.93)	271 (38.22)	—
C	1,186 (56.80)	160 (56.34)	659 (60.18)	367 (51.76)	—
S	158 (7.57)	41 (14.44)	75 (6.85)	42 (5.92)	—
SC	149 (7.14)	32 (11.27)	75 (6.85)	42 (5.92)	—
Survival time, Median (IQR)	6.00 (2.00,13.00)	12.00 (4.00, 27.00)	6.00 (2.00, 14.00)	4.00 (2.00, 9.00)	<0.001***
Vital status, n (%)	—	—	—	—	<0.001***
Alive	280 (13.41)	86 (30.28)	148 (13.52)	46 (6.49)	—
Cancer-specific death	1773 (84.91)	194 (68.31)	933 (85.21)	646 (91.11)	—
Other causes-specific death	35 (1.68)	4 (1.41)	14 (1.28)	17 (2.40)	—

*, two-sided *p* values <0.05; **, two-sided *p* values <0.01; ***, two-sided *p* values <0.001. AJCC, American Joint Committee on Cancer (seventh).

Treat, N, no treatment; C, chemotherapy alone; S, surgery alone; SC, surgery combined with chemotherapy.

### Univariate and Multivariate of OS in the Patients with Pancreatic Cancer Metastasis to Liver

As illustrated in Table 3, on the basis of the overall survival (OS), univariate analysis showed that the significant indicators were sex, grade, tumor size, AJCC N stage, histology and treatment in group age <52 years old; marital status, grade, tumor size, histology and treatment in group age 52–69 years old; and race, grade, AJCC N stage, histology and treatment in group age 69–84 years old.

**TABLE 3:** Univariate and multivariate of OS in the patients with pancreatic cancer metastasis to liver grouped by age at diagnosis.

	<52 years old				52–69 years old				69–84 years old			
Variables	Univariate analysis		Multivaraiate analysis		Univariate analysis		Multivaraiate analysis		Univariate analysis		Multivaraiate analysis	
	N	P Value	HR (95%CI)	P Value	N	P Value	HR (95%CI)	P Value	N	P Value	HR (95%CI)	P Value
Sex, n (%)	—	—	—	—	—	—	—	—	—	—	—	—
Female	119	—	—	—	438	—	—	—	343	—	—	—
Male	165	0.004^**^	1.28 (0.94–1.75)	0.112	657	0.126	1.10 (0.97–1.26)	0.15	366	0.113	0.87 (0.74–1.01)	0.075
Race, n (%)	—	—	—	—	—	—	—	—	—	—	—	—
White	207	—	—	—	865	—	—	—	579	—	—	—
Black	44	0.913	—	—	143	0.156	1.10 (0.90–1.33)	0.349	74	0.0645	1.00 (0.78–1.01)	0.976
Other	33	0.250	—	—	87	0.914	—	0.940	56	0.037*	1.22 (0.92–1.62)	0.176
Marital status, n (%)	—	—	—	—		—	—	—	—	—	—	—
Unmarried	87	—	—	—	186	—	—	—	60	—	—	—
Married	197	0.425	—	—	909	0.017*	0.85 (0.71–1.01)	0.063	0.940	0.0674	—	—
Grade, n (%)	—	—	—	—	—	—	—	—	—	—	—	—
Well differentiated	73	—	—	—	108	—	—	—	76	—	—	—
Moderately differentiated	82	<0.001***	2.52 (1.53–4.13)	<0.001***	414	<0.001***	3.16 (2.35–4,25)	<0.001***	260	<0.001***	2.07 (1.5–2.76)	<0.001***
Poorly differentiated	116	<0.001***	5.17 (3.14–8.52)	<0.001***	539	<0.001***	4.61 (3.43–6.19)	<0.001***	345	<0.001***	2.68 (2.02–3.57)	<0.001***
Undifferentiated	13	<0.001***	5.08 (2.24–11.53)	<0.001***	34	<0.001***	1.10 (0.82–1.47)	<0.001***	—	<0.001***	2.28 (1.43–3.65)	<0.001***
Tumor size, n (%)	—	—	—	—	—	—	—	—	—	—	—	—
<4.9 cm	164	—	—	—	544	—	—	—	401	—	—	—
4.9–7.4 cm	83	0.322	1.03 (0.95–1.81)	0.091	432	0.516	1.18 (1.03–1.36)	0.20*	69	0.744	—	—
>7.4 cm	37	0.008**	0.69 (0.41–1.16)	0.130	119	0.075	0.97 (0.78–1.20)	0.750	188	0.641	—	—
AJCC N, n (%)	—	—	—	—	—	—	—	—	—	—	—	—
N0	129	—	—	—	669	—	—	—	452	—	—	—
N1	1299	0.887	1.31 (0.75–1.42)	0.850	294	0.057	1.09 (0.94–1.27)	0.243	69	0.744	—	—
NX	26	<0.001^***^	0.69 (0.41–1.16)	0.162	132	0.003*	0.82 (0.66–1.03)	0.082	188	0.641	—	—
Histology, n (%)	—	—	—	—	—	—	—	—	—	—	—	—
Adenomas and adenocarcinomas	225	—	—	—	896	—	—	—	567	—	—	—
Ductal and lobular neoplasms	25	0.001**	1.69 (1.06–2.69)	0.027*	82	0.438	1.59 (1.24–2.04)	<0.001***	59	0.832	1.34 (1.01–1.79)	0.045*
Epithelial neoplasms	16	<0.001***	1.68 (0.93–3.01)	0.083	60	0.008*	1.48 (1.11–1.96)	0.007**	40	<0.001***	1.65 (1.18–2.31)	0.004*
Others	18	<0.001***	1.90 (1.13–3.21)	0.016	57	0.146	1.10 (O.82–1.47)	0.521	43	0.929	0.79 (0.57–1.10)	0.166
Treat n (%)	—	—	—	—	—	—	—	—	—	—	—	—
N	51	—	—	—	273	—	—	—	271	—	—	—
C	160	0.533	0.62 (0.41–0.94)	0.024*	659	<0.001***	0.43 (0.37–0.50)	<0.001***	367	<0.001***	0.54 (0.45–0.63)	<0.001***
S	41	<0.001***	0.16 (0.08–0.32)	<0.001***	75	<0.001***	0.15 (0.10–0.21)	<0.001***	42	<0.001***	1.65 (1.18–0.40)	<0.001***
SC	32	0.005**	0.35 (0.19–0.64)	<0.001***	88	<0.001***	0.16 (0.12–0.21)	<0.001***	29	<0.001***	0.33 (0.21–0.51)	<0.001***

*, two-sided *p* values <0.05; **, two-sided *p* values <0.01; ***, two-sided *p* values <0.001. AJCC, American Joint Committee on Cancer (seventh).

HR, hazard ratio.

CI, coincidence intervals. OS, overall survival.

Treat, N, no treatment; C, chemotherapy alone; S, surgery alone; SC, surgery combined with chemotherapy.

In multivariate analysis, we further observed the variables selected from univariate analysis (*p* < 0.2). Cox regression analysis was performed to compete hazard ratios and 95% confidence intervals. In the three groups, tumor grade was all associated with poor overall survival, and surgery alone (S) was the best treatment option for the overall survival of patients.

Using AJCC N0 stage as reference, AJCC N1 stage (*p* = 0.020, HR = 1.18, 95%CI, 1.03–1.36) in group age 52–69 years and AJCC NX stage (*p* = 0.039, HR = 1.33, 95%CI, 1.01–1.74) in group age 69–84 years old were indicated to be associated with poor overall survival, while in group age <52 years old, AJCC N stage was not correlated with the prognosis. Choosing adenomas and adenocarcinomas as reference in histological types, in addition to ductal and lobular neoplasms (age <52 years, *p* = 0.027, HR = 1.69, 95%CI, 1.06-2.69; age 52–69 years old, *p* < 0.001, HR = 1.59, 95%CI, 1.24-2.04; age 69–84 years old, *p* = 0.045, HR = 1.34, 95%CI, 1.01-1.79) in the three groups, other histological types (*p* = 0.016, HR = 1.90, 95%CI, 1.13-3.21) in group age <52 years old, and epithelial neoplasms (age 52–69 years old, *p* = 0.007, HR = 1.48, 95%CI, 1.11-1.96; age 69–84 years old, *p* = 0.004, HR = 1.65, 95%CI, 1.18-2.31) in the other two groups (age >52 years old) were associated with a poor overall survival.

### Univariate and Multivariate of CSS in the Patients with Pancreatic Cancer Metastasis to Liver

As illustrated in Table 4, on the basis of the cancer-specific survival (CSS), univariate analysis showed that the significant indicators were sex, grade, histology and treatment methods in group age <52 years old; grade, histology and treatment methods in group age 52–69 years old; and race, grade, AJCC T stage and treatment methods in group age 69–84 years old.

**TABLE 4:** Univariate and multivariate of CSS in the patients with pancreatic cancer metastasis to liver grouped by age at diagnosis.

Variables	<52 years old				52–69 years old				69–84 years old			
	Univariate		Multivariate		Univariate		Multivariate		Univariate		Multivariate	
	N	*p* value	HR (95%CI)	*p* value	N	*p* value	HR (95%CI)	*p* value	N	*p* value	HR (95%CI)	*p* value
Sex, n (%)												
Female	73	—	—	—	365	—	—	—	317	—	—	—
Male	121	0.045*	1.08 (0.78–1.48)	0.645	568	0.274	—	—	329	0.428	—	—
Race, n (%)												
White	142	—	—	—	735	—	—	—	527	—	—	—
Black	31	0.308	—	—	127	0.814	—	—	65	0.208	1.08 (0.83–1.40)	0.576
Other	21	0.796	—	—	71	0.253	—	—	54	0.028*	1.42 (1.07–1.90)	0.017*
Marital status, n (%)												
Unmarried	56	—	—	—	158	—	—	—	55	—	—	—
Married	138	0.706	—	—	775	0.073	0.88 (0.74–1.04)	0.14	591	0.975	—	—
Grade, n (%)												
Well differentiated	25	—	—	—	54	—	—	—	58	—	—	—
Moderately differentiated	53	<0.001***	4.05 (2.25–7.29)	<0.001***	353	0.024*	1.61 (1.20–2.16)	0.002**	237	<0.001***	1.99 (1.48–2.68)	<0.001***
Poorly differentiated	106	<0.001***	5.26 (2.97–9.33)	<0.001***	497	<0.001***	2.29 (1.71–3.06)	<0.001***	325	<0.001***	2.50 (1.86–3.36)	<0.001***
Undifferentiated	10	<0.001***	9.43 (3.76–23.67)	<0.001***	29	0.003**	2.00 (1.25–3.20)	0.004**	26	0.002**	2.14 (1.33–3.46)	0.002**
Tumor size, n (%)												
<4.9 cm	113	—	—	—	576	—	—	—	414	—	—	—
4.9–7.4 cm	64	0.601	—	—	260	0.514	—	—	170	0.651	—	—
>7.4 cm	17	0.854	—	—	97	0.928	—	—	62	0.255	—	—
AJCC N, n (%)												
N0	86	—	—	—	468	—	—	—	362	—	—	—
N1	85	0.601	1.82 (1.29–2.56)	<0.001***	359	0.491	—	—	223	0.041*	0.94 (0.79–1.12)	0.47
NX	23	0.077	1.17 (0.72–1.92)	0.523	106	0.338	—	—	61	0.055	1.13 (0.86–1.50)	0.382
Histology, n (%)												
Adenomas and adenocarcinomas	139	—	—	—	753	—	—	—	513	—	—	
Ductal and lobular neoplasms	23	0.764	1.05 (0.66–1.68)	0.832	75	0.309	1.19 (0.92–1.54)	0.178	55	0.639	1.15 (0.85–1.56)	0.353
Epithelial neoplasms	14	0.024*	1.17 (0.63–2.17)	0.622	55	0.009**	1.38 (1.04–1.84)	0.026*	38	0.002**	1.43 (1.01–2.02)	0.042*
Others	18	0.016*	1.35 (0.80–2.27)	0.264	50	0.136	1.18 (0.88–1.58)	0.268	40	0.834	0.84 (0.60–1.17)	0.293
Treat, n (%)												
N	36	—	—	—	245	—	—	—	252	—	—	—
C	131	0.036*	0.44 (0.29–0.69)	<0.001***	601	<0.001***	0.40 (0.35–0.47)	<0.001***	340	<0.001***	0.48 (0.40–0.57)	<0.001***
S	9	0.008**	0.22 (0.09–0.52)	<0.001***	33	<0.001***	0.30 (0.21–0.44)	<0.001***	31	<0.001***	0.40 (0.26–0.60)	<0.001***
SC	18	<0.001***	0.17 (0.08–0.33)	<0.001***	54	<0.001***	0.22 (0.16–0.30)	<0.001***	23	<0.001***	0.44 (0.28–0.69)	<0.001***

*, two-sided *p* values <0.05; **, two-sided *p* values <0.01; ***, two-sided *p* values <0.001. AJCC, American Joint Committee on Cancer (seventh).

HR, hazard ratio.

CI, coincidence intervals. CSS, cancer-specific survival.

Treat, N, no treatment; C, chemotherapy alone; S, surgery alone; SC, surgery combined with chemotherapy.

Using well differentiated grade as reference, multivariate analysis in Table 4 indicated tumor grade was associated with poor overall survival at different ages. In addition, treatment (S, C, SC) was associated with better cancer-specific survival in all three groups compared with no treatment. Notably, in group age 69–84 years old, surgery alone (S, *p* < 0.001, HR = 0.40, 95%CI, 0.26-0.60) was the optimal treatment, whereas surgery combined with chemotherapy (SC, group age <52 years old, *p* < 0.01, HR = 0.17, 95%CI, 0.08-0.33; group age 52–69 years old, *p* < 0.001, HR = 0.22, 95%CI, 0.16-0.30) was the best option in the other groups.

When using AJCC N0 as reference, patients with AJCC N1 stage (*p* < 0.001, HR = 1.82, 95%CI, 1.29-2.56) had a poor prognosis only in group age <52 years old. And epithelial neoplasms (age 52-69, *p* = 0.026, HR = 1.38, 95%CI, 1.04-1.84; age 69–84 years old, *p* = 0.042 HR = 1.43, 95%CI, 1.01-2.02) were associated with a poor cancer-specific survival only in group age >52 years old when using adenomas and adenocarcinomas as reference. Additionally, in group age 69–84 years old, other racial patients (*p* = 0.017, HR = 1.42, 95%CI, 1.07-1.90) had a worse prognosis.

## Discussion

It was reported that 90% cancer-related deaths resulted from metastasis of the primary tumor. The formation of local infiltrates and metastases are clinically most relevant to the progression of cancer (Christofori, 2006). Organ damage due to growth-related lesions, paraneoplastic syndromes, or treatment complications was significantly associated with morbidity and mortality of metastatic disease (Steeg, 2006). In general, cancer metastasis can be divided into different stages from local invasion, intravasation, survival in circulation, extravasation, finally to colonization and metastasis (Hanahan and Weinberg, 2011). The unique biological characteristics of the liver make it a vulnerable site for tumor metastasis: 1) structural and hemodynamic features - characteristic microcirculation in the liver makes it easier for diffuse tumor cells carried in the blood to enter. In addition, molecules on the surface of hepatic nonparenchymal cells (NPCs) lining the hepatic capillaries contribute to the adhesion and retention of circulating tumor cells. The pore on the hepatic sinusoidal endothelial cell (LSECs) facilitates the tumor cells to enter the basement membrane directly; 2) regenerative capabilities—the cellular tissue remodeling mechanism involved in self-renewal and reconstruction that promotes intratumoral stroma and blood vessel formation through signals generated by tumor cells, creating an enabling environment for survival and growth; 3) regional immunosuppression—the general foreign body reaction is reduced to limit potential damage to the liver, resulting in a relatively tolerant microenvironment that allows for the survival and growth of foreign tumor cells (Vidal-Vanaclocha, 2011; Clark et al., 2016).

Pancreatic cancer is the fourth leading cause of cancer-related death worldwide, and its main metastatic site is liver (Stott et al., 2010). Studies have shown that, in addition to smoking, a family history of pancreatic cancer, black race, diabetes, and increased body mass index were also predictors of pancreatic cancer mortality (Coughlin et al., 2000). A lack of early signs and symptoms, as well as high aggressiveness, leads to a low survival rate. The prognosis of patients with pancreatic cancer is closely related to tumor stage and tumor grade/aggressiveness (Bolm et al., 2015) that can only be evaluated by biopsy or surgery. Our present data showed tumor grade was also a significant predictor of overall survival and cancer-specific survival in patients with liver metastasis, independent of age at diagnosis (Table 3, Table 4). In addition, 85% of the histology types of pancreatic cancer are ductal adenocarcinoma of the pancreas (PDAC) (Ryan et al., 2014; Hogendorf et al., 2018). For patients with PDAC, younger age, male sex, larger tumor size, low ALT level and high CA 19-9 level could predict unexpected distant metastasis (Liu et al., 2018). Histologically, pancreatic adenocarcinoma accounts for the largest proportion in pancreatic cancer (Simard et al., 2012), accompanied with the worst prognosis, and the most common site of metastasis is liver (Lemke et al., 2013; Deeb et al., 2015; Kumar et al., 2015), which is consistent with our results (Table 2). Our data suggested that most histologic types of pancreatic metastases to liver were adenocarcinomas. The prognosis of patients with pancreatic cancer with liver metastasis was poorer than that of patients with distant lymph node metastasis or lung metastasis. The factors predicting the better prognosis included age<65 years, white race, being married, female sex and surgery treatment (Oweira et al., 2017). Furthermore, our study showed that the younger the age, the higher the overall survival rate of patients with pancreatic cancer with liver metastasis (Figure 4). In addition, we found differences in prognostic factors among the groups after grouping by age at diagnosis. Histologically, compared with pancreatic adenocarcinoma, ductal and lobular neoplasms and epithelial neoplasms were associated with poor overall survival in the group age >52 years old, while the latter were not correlated with the prognosis in group age <52 years old. In the multivariate regression analysis, histological type was a significant predictor for cancer-specific survival only for patients diagnosed at age >52 years old. AJCC N1 stage with significance in predicting poor overall survival only in group age 52–69 years old, and predicting poor cancer-specific survival only in group age <52 years old. (Table 3, Table 4).

At present, the only treatment for pancreatic cancer is surgery, and adjuvant therapy based on chemotherapy can improve the survival rate (McGuigan et al., 2018). For elderly patients (age>80 years old), postoperative adjuvant chemotherapy is critical to the prognosis (Sho et al., 2016). Surgery is limited to patients with localized disease, and metastatic spread is often considered a contraindication to resection, regardless of whether it is observed synchronously or ectopic (Seufferlein et al., 2012). However, metastatic excision or local treatment is occasionally performed in centers around the world based on individual clinical experience, and there is no objective evidence to guide treatment methods taking into account patient choice or metastatic spread (Gleisner et al., 2007; Shrikhande et al., 2007; De Jong et al., 2010; Nentwich et al., 2012; Edwards et al., 2013). In general, palliative chemotherapy with FOLFIRONOX (mFOLFIRINOX with 5-fluorouracil) is the preferred chemotherapy regimen for metastatic pancreatic cancer (McGuigan et al., 2018). Despite this, T. Hackert (Hackert et al., 2017) proved that resection of liver or interaortocaval lymph nodes (ILN) metastases could be superior to palliative treatment for pancreatic cancer patients with metastasis. Mitsuka et al. (2020) found that the median survival time was significantly improved for patients diagnosed between 44 and 83 years old who underwent liver resection or pancreatectomy. Other study (Warschkow et al., 2020) showed that lymphadenectomy had only 18% direct effect on improved overall survival, while 82% of its effect were mediated by other factors like treatment at high-volume hospitals and adjuvant chemotherapy for patients whose median age were 66 years. However, the analysis on differences among different ages of patients is scarce. As we know, there is insufficient evidence that the efficacy of different therapies in patients with metastatic pancreatic cancer is age-related. Our data showed chemotherapy alone was the most important prognostic factor for patients who diagnosed at younger age, and age of diagnosis was the most prognostic factor for patients diagnosed at an older age. For the diagnosis of pancreatic cancer at all ages, surgery was the best treatment method to improve the overall survival rate of patients with pancreatic cancer with liver metastasis (Table 3). Considering the tumor-specific survival rate, surgery combined with chemotherapy is the best choice for patients under 69 years of age at the time of diagnosis, while surgery alone is the best choice for patients aged 69–84 years at the time of diagnosis. In addition, surgery alone and combined chemotherapy were significantly superior to chemotherapy alone in terms of overall survival and tumor-specific survival (Table 3, Table 4). Interestingly, in a case report (Katsura et al., 2019), after the combination therapy of pancreatoduodenectomy and chemotherapy, a 66-year-old patient with pancreatic ductal carcinoma metastasis to liver showed the disappearance of liver metastasis and without other new metastasis. This case report partially confirms the conclusion from our analysis that surgery alone was the optimal treatment in group age>69 years old, while surgery combined with chemotherapy was the best option in the other groups. Both surgical treatment and chemotherapy cause damage to human bodies. Especially, the elderly can hardly bear the double blow, as surgical treatment and chemotherapy both exerting in therapy. In addition, chemotherapy is often accompanied with many side effects. The analyzed data in this manuscript demonstrates that surgical treatment alone is superior to surgery plus chemotherapy in patients older than 69 years of age. It suggests that surgery should be a priority for the older population (age>69) with pancreatic cancer metastasis to liver. Certainly, clinical treatment selection depends on the multiple assessment of patient, and this manuscript provides an epidemiological reference for the selection of clinical treatment.

Although the SEER database provides a large amount of clinical data, there are still many limitations in our research. First, we need to further conduct a follow-up clinical trial to verify this result. Second, we did not include patients undergoing radiotherapy because of the small number of cases, and we need to compare the effects of radiotherapy, chemotherapy and surgery on prognosis. Finally, the sequence of chemotherapy and surgery, the diverse methods of surgery and chemotherapy can be further studied.

## Conclusion

In this population-based analysis, we found the main primary sites of metastatic liver cancer are lung and brunchu, sigmoid colon and pancreas. Furthermore, we explored the prognostic factors of pancreatic cancer metastasis to liver grouped by age at diagnosis. Tumor grade, histology and treatment are valid prognostic factors in all age groups. Additionally, gender and AJCC N stage in age<52 years old, while race and AJCC N stage in age>69 years old were predictors. Surgery alone was the optimal treatment in group age>69 years old, whereas surgery combined with chemotherapy was the best option in the other groups. In conclusion, these findings would help to choose better treatment for patients with metastatic liver cancer.

## Data Availability

Publicly available datasets were analyzed in this study. This data can be found here: https://seer.cancer.gov/.The data was from the National Cancer Institute’s Surveillance, Epidemiology, and End Results (SEER) program between 2004 and 2015.

## Abbreviations

AJCC, American Joint Committee on Cancer (seventh); CI, coincidence intervals; CSS, cancer-specific survival; HR, hazard ratio; OS, overall survival; SEER, Surveillance Epidemiology and End Results.
